# Search for unknown neural link between the masticatory and cognitive brain systems to clarify the involvement of its impairment in the pathogenesis of Alzheimer’s disease

**DOI:** 10.3389/fncel.2024.1425645

**Published:** 2024-06-27

**Authors:** Youngnam Kang, Hiroki Toyoda, Mitsuru Saito

**Affiliations:** ^1^Department of Behavioral Physiology, Osaka University Graduate School of Human Sciences, Osaka, Japan; ^2^Department of Oral Physiology, Osaka University Graduate School of Dentistry, Osaka, Japan; ^3^Department of Oral Physiology, Graduate School of Medical and Dental Sciences, Kagoshima University, Kagoshima, Japan

**Keywords:** Alzheimer’s disease, locus coeruleus, mesencephalic trigeminal nucleus, neurotrophic factor-3 (NT-3), 3,4-dihydroxyphenylglycolaldehyde (DOPEGAL)

## Abstract

Brain degenerations in sporadic Alzheimer’s disease (AD) are observed earliest in the locus coeruleus (LC), a population of noradrenergic neurons, in which hyperphosphorylated tau protein expression and β-amyloid (Aβ) accumulation begin. Along with this, similar changes occur in the basal forebrain cholinergic neurons, such as the nucleus basalis of Meynert. Neuronal degeneration of the two neuronal nuclei leads to a decrease in neurotrophic factors such as brain-derived neurotrophic factor (BDNF) in the hippocampus and cerebral cortex, which results in the accumulation of Aβ and hyperphosphorylated tau protein and ultimately causes neuronal cell death in those cortices. On the other hand, a large number of epidemiological studies have shown that tooth loss or masticatory dysfunction is a risk factor for dementia including AD, and numerous studies using experimental animals have also shown that masticatory dysfunction causes brain degeneration in the basal forebrain, hippocampus, and cerebral cortex similar to those observed in human AD, and that learning and memory functions are impaired accordingly. However, it remains unclear how masticatory dysfunction can induce such brain degeneration similar to AD, and the neural mechanism linking the trigeminal nervous system responsible for mastication and the cognitive and memory brain system remains unknown. In this review paper, we provide clues to the search for such “missing link” by discussing the embryological, anatomical, and physiological relationship between LC and its laterally adjoining mesencephalic trigeminal nucleus which plays a central role in the masticatory functions.

## 1 Introduction

Brain degeneration in sporadic Alzheimer’s disease (AD) is recognized earliest in the locus coeruleus (LC), a population of noradrenergic (NA-ergic) neurons, where hyperphosphorylated tau protein is expressed and β-amyloid (Aβ) gradually accumulates, resulting in up to 80% cell death of LC neurons (Vijayashankar and Brody, 1979; Bondareff et al., 1982). In sporadic AD, cell death also occurs in basal forebrain (BF) cholinergic neurons (BFC neurons) (Whitehouse et al., 1981); especially up to 75% or more cell death in the nucleus basalis of Meynert (NBM) (Whitehouse et al., 1982). Degeneration or cell death of the two nuclei leads to a reduction of acetylcholine (ACh) and NA inputs projecting to the hippocampus and cerebral cortex. Subsequently, neurotrophic factors such as nerve growth factor (NGF) or brain-derived neurotrophic factor (BDNF) are reduced in the hippocampus and cerebral cortex, which causes the accumulation of Aβ and the generation of hyperphosphorylated tau protein, ultimately leading to neuronal cell death and brain atrophy in the hippocampus and cerebral cortex (Counts and Mufson, 2010; Liu et al., 2015; Ballinger et al., 2016; Chen et al., 2018). Thus, in addition to Aβ and hyperphosphorylated tau protein, neurotrophic factors such as NGF, BDNF, and neurotrophin-3 (NT-3), and their receptors, TrkA/B/C, have been shown to be involved in the onset and progression of AD (Chen et al., 2018). The cell deaths of the two core neuronal nuclei of LC and NBM (BF) which cause dementia were both established by 1982 in AD patients (Bondareff et al., 1982; Whitehouse et al., 1982), and subsequent studies based on these findings have revealed details of the crucial roles of LC and BF in learning, memory and cognitive functions (Ridley et al., 1986; Aigner et al., 1987; Aston-Jones et al., 1991; Aston-Jones and Cohen, 2005).

On the other hand, a large number of epidemiological studies have revealed an involvement of masticatory dysfunction in inducing dementia and AD (Kondo, 1990; Isse et al., 1991; Gatz et al., 2006; Ikebe et al., 2018). Consistent with the epidemiological studies, numerous studies in rats and mice showed that masticatory dysfunction caused cell death of NBM neurons (Terasawa et al., 2002), decreased ACh (Kato et al., 1997; Makiura et al., 2000) and BDNF (Lee et al., 2008; Furukawa et al., 2022), and subsequently caused accumulation of Aβ in the hippocampus and cerebral cortex (Ekuni et al., 2011, 2013), resulting in synaptic dysfunction and cell death in the hippocampus and cortex (Taslima et al., 2022). It has also been reported that learning and memory functions are impaired as a consequence of masticatory dysfunction (Kato et al., 1997; Yamazaki et al., 2008; Hirai et al., 2010; Ekuni et al., 2013; De Cicco et al., 2017). In addition to these studies in experimental animals, a significant negative correlation between the number of remaining teeth and tau degeneration/pathology in the LC and hippocampus was recently reported in a positron emission tomography study in human AD patients (Matsumoto et al., 2023). However, it remains unclear how masticatory dysfunction causes the decrease and/or accumulation of the key molecules, and the neural mechanism linking the trigeminal nervous system responsible for occlusal mastication function and the brain system of cognition and memory remains unclear.

In this review article, we will discuss the embryological, anatomical, and physiological relationships between the BF/LC which play key roles in the pathogenesis of AD and the mesencephalic trigeminal nucleus (MTN) which plays a central role in occlusal and masticatory function, in order to facilitate the discovery of the missing link.

## 2 Current status of the research on the relationship between the trigeminal nervous system and AD studied using transgenic AD model mice

There is a very limited number of studies that investigated the relationship between the trigeminal nervous system and AD using transgenic AD mice. Recently, studies using 5xFAD mice, a type of AD model mice, revealed that at the age of 5 months Aβ is prominently accumulated in the trigeminal motor nucleus, resulting in cell death and atrophy of myofibers in the jaw-closing muscle (Kim et al., 2021). This finding indicates that masticatory function may be impaired following the development of AD. However, such degeneration in the trigeminal motor nucleus has not been observed in the postmortem brains of human AD patients (Giess and Schlote, 1995; Parvizi et al., 2001; Uematsu et al., 2018). Furthermore, even in 9- to 15-month-old 5xFAD mice, cell death of only 10–20% of BFC neurons was observed (Yan et al., 2018), in contrast to the cell death of more than 75% of NBM neurons in human AD. Moreover, neither accumulation of Aβ and expression of hyperphosphorylated tau protein nor cell death of NAergic neurons were observed in the LC of 5-month-old 5xFAD mice, in contrast to human AD. Therefore, it is not easy to evaluate the relationship between human AD and cell death in the trigeminal motor nucleus due to Aβ accumulation demonstrated in 5-month-old 5xFAD mice.

The endogenous mechanism of cell death of LC neurons, which triggers the pathogenic cascade of AD (see below), has been well established by many studies (Burke et al., 1999; Kang et al., 2020, 2022), and it has also been established that hyperphosphorylated tau protein expression and Aβ accumulation occur prior to other regions of the brain (Mather and Harley, 2016). In a study using 4- to 5-month-old transgenic 3xTG-AD mice, it was observed that the Aβ accumulated in MTN neurons leaked out and diffused extracellularly when cell death of MTN neurons was induced by tooth extraction, triggering an inflammatory response in surrounding regions. Consequently, this caused cell death of adjacent LC neurons, resulting in memory impairment (Goto et al., 2020). However, this is not consistent with the endogenous mechanism of cell death of LC neurons in AD (see below). In this study (Goto et al., 2020), neither hyperphosphorylated tau protein nor Aβ was detected in LC neurons themselves in contrast to human AD, suggesting that the endogenous mechanism of cell death of LC neurons in AD did not function. Thus, because the cell death of LC neurons appeared to be the result of a spillover of an inflammatory response, it could be an etiologic factor for other types of dementia, but not for AD. Therefore, it is not easy to find a rationale for such studies and it may be more reasonable to test the hypothesis that the endogenous mechanism of LC cell death is accelerated by the depletion of trophic factors paracrine-secreted from the adjacent MTN, in order to examine the possible involvement of MTN neurons in AD pathogenesis. In 5-month-old 5xFAD mice, Aβ accumulated not only in the trigeminal motor nucleus but also in the MTN, causing cell death, but no effect on LC was observed (Kim et al., 2021). In the postmortem brains of AD patients, accumulation of hyperphosphorylated tau protein and Aβ in the LC has been observed, whereas no accumulation of Aβ in the MTN has been observed (Tables 6 and 7 in Giess and Schlote, 1995; Parvizi et al., 2001).

Thus, the function of the trigeminal neural circuitry influencing the LC and BF has not yet been revealed and the neural linkage between the masticatory brain function and the cognitive brain function is still missing.

## 3 Is Aβ the most upstream signaling factor responsible for the development of AD?

Postmortem brain autopsies of senile people who did not show cognitive impairment and those who had been diagnosed with AD revealed that most of the senile plaques in both groups were Aβ42-immunopositive, but the proportion of senile plaques that were also Aβ40-immunopositive was slightly higher in the AD group than in the non-AD group (25% vs. 13%) (Fukumoto et al., 1996). Based on these observations, it was concluded that there are no significant qualitative differences in the pattern of Aβ types of senile plaques, suggesting the presence of preclinical AD (Fukumoto et al., 1996). Recently, however, it has become known that Aβ42-positive diffuse senile plaques are more common in normal senile patients, while Aβ40-positive typical senile plaques with core formation are more common in AD patients than in normal senile patients, along with diffuse senile plaques (Thal et al., 2006). Furthermore, it has recently been proposed that the N-terminal pyroglutamylation and hydrophobicity of Aβ42 enhances the aggregation of Aβ42-positive diffuse senile plaques as well as the formation of Aβ40-positive senile plaques, which play a critical role in the priming and maturation of pathogenic senile plaque formation (Michno et al., 2019). However, a clinical trial using the Aβ vaccine AN-1972 reported that the progression of cognitive dysfunction itself was not inhibited by the vaccine although senile plaques themselves were significantly reduced (Gilman et al., 2005). In addition, it is known that metabolites of NA induce Aβ production in LC neurons, as described below (Burke et al., 1999). Therefore, it remains to be determined whether Aβ is truly the most upstream signaling factor responsible for AD. Nevertheless, it is known that Aβ accumulation begins 15–20 years before cognitive impairment and leads to the generation of hyperphosphorylated tau protein and neurofibrillary tangle (NFT), and that people with senile plaque accumulation or decreased cholinergic nerve fibers are at high risk of developing AD (preclinical AD) even if they do not show cognitive impairment (Jacobs et al., 1995; Beach et al., 1997; Jack et al., 2010). Therefore, Aβ is still considered to be involved in the most upstream process of AD pathogenesis.

In 1987, a gene which is involved in the production of Aβ in familial AD patients was identified (Goldgaber et al., 1987), and the mutations in normal genes and the extent to which Aβ is overproduced compared to normal genes were also elucidated (Citron et al., 1992). Since then, more than 200 types of transgenic mice in which DNAs encoding amyloid precursor protein and presenilin were mutated have been developed to overproduce Aβ and hyperphosphorylated tau protein. The neurotoxicity of Aβ and NFTs has been investigated using such genetically engineered mice. Aβ causes membrane potential depolarization (Good et al., 1996; Blanchard et al., 1997), and triggers a neurotoxic cascade of responses including oxidative stress, mitochondrial depolarization, and apoptosis (Mattson, 2006). Alternatively, it activates microglia and induces neuroinflammation (Olmos-Alonso et al., 2016).

However, even in these studies using transgenic AD model mice, the pathogenesis of AD remains to be established, and it must be questioned whether Aβ is truly responsible for the upstream processes. In fact, transgenic mice overproducing Aβ or NFTs failed to induce marked cell death of NBM neurons, in contrast to human AD (Yan et al., 2018). To clarify the role of BFC neurons such as in NBM in AD pathogenesis, selective lesioning of BFC neurons was made using the immunotoxin p75-saporin in such transgenic mice. The lesioning of BFC neurons resulted in an earlier appearance of Aβ accumulation and memory impairment in the cortex and hippocampus (Laursen et al., 2013; Ramos-Rodriguez et al., 2013). It was also found that lesioning of BFC neurons caused a decrease in neurotrophic factors in the cortex and hippocampus, resulting in enhanced production of Aβ, but not NFT generation (Turnbull and Coulson, 2017).

It has also been reported that activation of nicotinic receptors in cortical pyramidal cells by the activity of BFC neurons may cause the production of neurotrophic factors in pyramidal cells (Hotta et al., 2009). Thus, the lesioning of BFC neurons promoted the accumulation of Aβ, but transgenic AD model mice alone, which overproduce Aβ or NFT, did not cause early and pronounced cell death of BFC neurons, in contrast to human AD (Yan et al., 2018). On the other hand, it has been reported in human studies using MRI that degeneration of BFC neurons precedes the degenerative expansion in the cerebral cortex and can predict its extent in AD patients (Schmitz et al., 2016). Since the development of various transgenic AD model mice, there has been much debate as to whether AD begins in the BF or the hippocampus, but by 2016, many studies had concluded that AD begins in the BF (Ballinger et al., 2016; Schmitz et al., 2016), which also put a question on the Aβ-most upstream theory and revealed the importance of BFC neurons. These results suggest that the decrease in neurotrophic factors due to cell death of BFC neurons may be a more upstream process in the pathogenesis of AD than Aβ production (Hotta et al., 2009; Ramos-Rodriguez et al., 2013; Ballinger et al., 2016; Turnbull and Coulson, 2017).

## 4 Functional effects of the LC-NA system on the cerebral cortex

The LC-NA system plays an important role in determining cognitive function in old age; the LC is often the first brain region in which AD-related pathology is found, with most people showing at least some tau pathology by their mid-20s (Mather and Harley, 2016). In AD, extensive cell death in the LC precedes that of the cortex and hippocampus (Mather and Harley, 2016; Schmitz et al., 2016).

NA, a neurotransmitter of the LC, activates β adrenergic receptors (ARs) in cortical and hippocampal pyramidal cells, causing paracrine secretion of NGF and BDNF, and inhibiting oxidative stress, mitochondrial depolarization, and caspase activation caused by Aβ (Counts and Mufson, 2010). NA also suppresses neuroinflammatory responses in the brain; microglia stimulated by NA suppress Aβ-induced production of cytokines and chemokines and increase microglial migration and phagocytosis of Aβ (Heneka et al., 2010). Furthermore, it has been reported that NA deficiency induces increased Aβ deposition in the cerebral cortex of AD model mice (Heneka et al., 2010). Based on the discovery of significant LC cell death in senile AD and the neuroprotective effects of noradrenaline, the “Noradrenergic Theory of Cognitive Reserve” was proposed (Robertson, 2013). The theory proposed that upward regulation of the LC-NA system through lifelong education and learning stimulates and improves cognitive function and contributes to cognitive reserve to prevent neurodegeneration.

## 5 MTN neuronal activity may influence survival and maintenance of LC neurons

**Figure 1:**
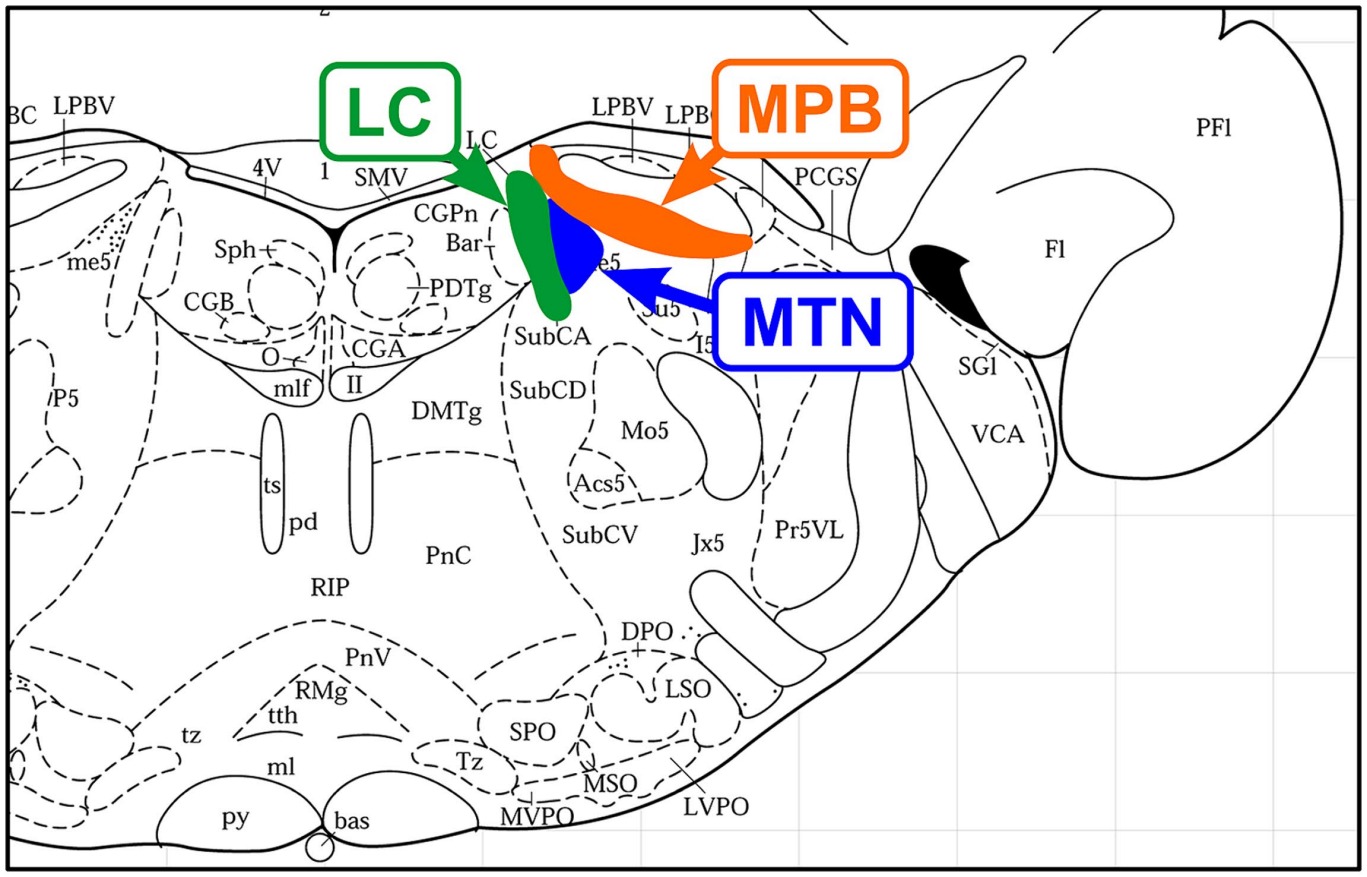
Location of nerve nuclei in a coronal section of the rat brainstem (Bregma, −9.68 mm; Paxinos and Watson, 1996). From medial to lateral, Locus coeruleus (LC), mesencephalic trigeminal nucleus (MTN), and medial part of the parabrachial nucleus (MPB) are located adjacent to each other.

Interestingly, it is reported that the differentiation and development of LC in the late embryonic period are dependent on the presence of adjacent MTN in which Onecut factors play not only a cell-autonomous role in its differentiation and development but also play a non-cell autonomous role in LC development (Espana and Clotman, 2012). Thus, it was suggested that the presence of the MTN would be required for the maintenance of the noradrenergic phenotype of the LC neurons. Therefore, it is possible that MTN, which is supposed to be located in the ganglion outside the brain, is located adjacent to the LC (Figure 1). Even after maturation, viability of LC neurons may be maintained by the function of a trophic factor, NT-3, that is paracrine secreted from MTN depending on muscle spindle activity. The secretory molecule of NT-3 is normally produced by muscle spindles (Ernfors et al., 1994; Tessarollo et al., 1994), and is released activity-dependently to bind to TrkC receptors expressed on peripheral fiber endings of primary sensory neurons innervating muscle spindles, and is taken up into nerve endosomes and transported to the cell body of MTN neurons via retrograde axonal transport (Friedel et al., 1997).

Among all the primary sensory neurons, MTN neuron is only exceptionally located in the brain and medially adjoins LC (Copray et al., 1990; Takahashi et al., 2010; Figure 1). As such, they receive a variety of synaptic inputs including abundant NA-like projections from the LC (Takahashi et al., 2010). MTN neurons are also known to undergo somatic exocytosis during neuronal activity (Zhang et al., 2012). Therefore, if NT-3 is paracrine secreted from the cell body of MTN neurons and taken up by binding to TrkC receptors (Merlio et al., 1992; Sandell et al., 1994, 1998) expressed on nerve endings projecting from the LC to the MTN, NT-3 may be useful for the maintenance of function and/or the survival of LC neurons and may be a mechanism to prevent cell death as reported previously (Arenas and Persson, 1994).

**Figure 2:**
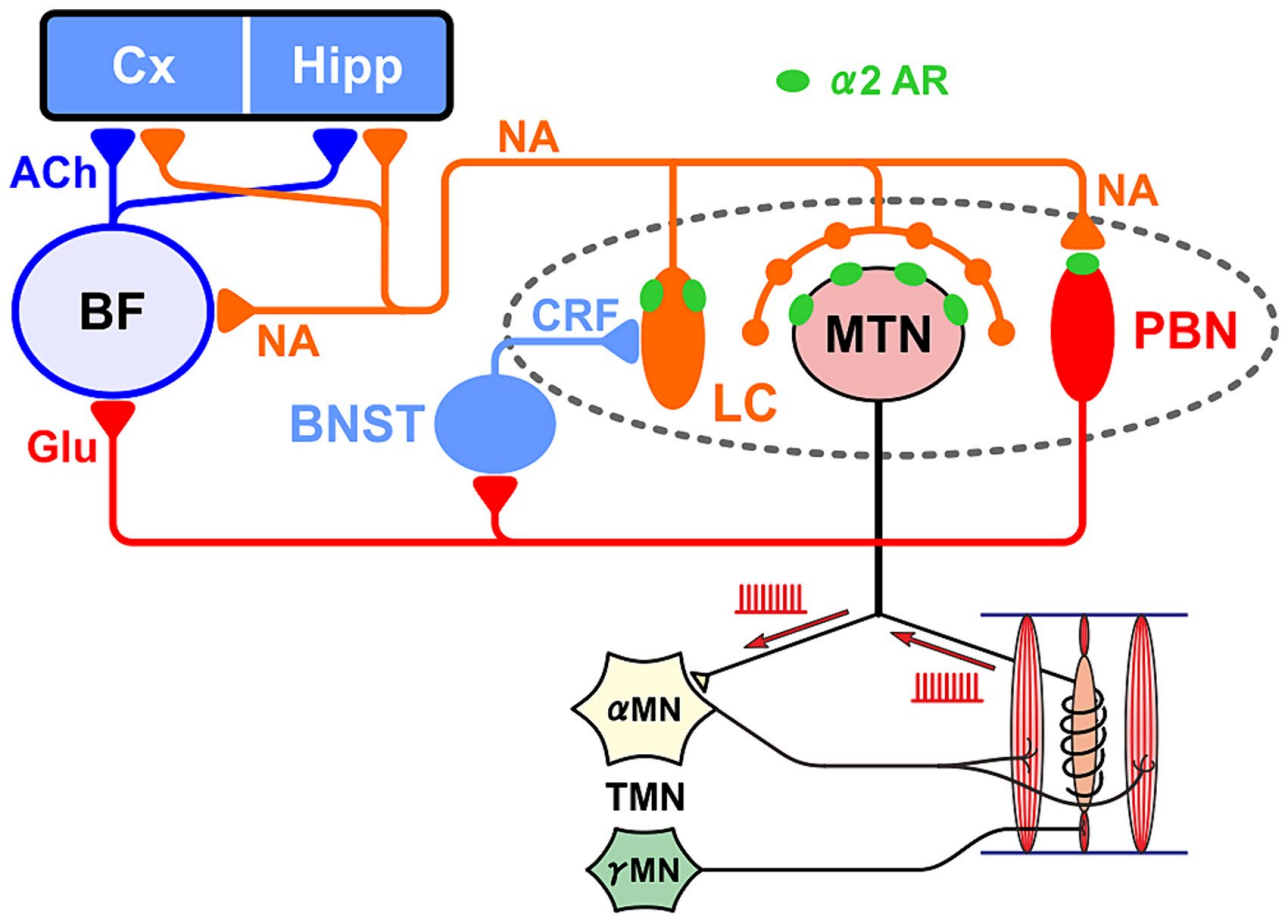
Relationship between the LC-MTN-PBN neural circuit and the basal forebrain (BF), cortex (Cx) and hippocampus (Hipp). LC projections to MTN and PBN exert excitatory and inhibitory effects through the activation of α2A adrenergic receptor (AR), respectively. LC also projects to BF. Glutamatergic PBN is the core nucleus of the ascending arousal system, and activates MN and the bed nucleus stria terminalis (BNST) that activates LC. The activity of γ-motoneurons (γMN) produces neurotrophic factor NT-3 in muscle spindles, which is transported retrogradely to MTN and subsequently can be paracrine secreted from MTN. The viability of Cx and Hipp cells is maintained by noradrenergic (NA) and cholinergic (ACh) inputs, while conversely AD develops due to the impairment of LC and BF.

The parabrachial nucleus (PBN), along with the pedunculopontine tegmental nucleus, plays a central role in the arousal system through projections to the thalamus and BF as the center of the reticular activation system (Fuller et al., 2011). It is also interesting to note that the medial PBN (MPB) laterally adjoins the MTN. The impairment of these neural circuits between the LC-MTN-MPB (Figure 2) may be involved in the pathogenesis of AD, and further studies are warranted.

Sensory information in the trigeminal nervous system plays an important role in the activation of the arousal system. On the other hand, the loss of multiple teeth is considered to cause Alzheimer’s-type learning and memory deficits as a result of reduced sensory information. However, it has also been reported that elevation of the height of the occlusion can cause learning and memory impairment (Piancino et al., 2019; Toyoda et al., 2023), and it has been proposed that the cause is not a decrease or increase in input information, but the stress caused by the dysfunction of masticatory motor control system due to the error or decrease in such sensory information (Budtz-Jorgensen, 1981; Piancino et al., 2019, 2020; Toyoda et al., 2023).

## 6 How are LC neurons involved in occlusal masticatory function?

The firing activity of LC neurons transiently increases in response to panic and mental stress (Valentino and Foote, 1988; Curtis et al., 1997), which is known to be caused by the activation of corticotropin-releasing factor (CRF) receptors by CRF (Van Bockstaele et al., 1998; Koob, 1999) secreted in response to stress from the bed nucleus of the stria terminalis (BNST), hypothalamus and amygdala (Valentino et al., 1983; McCall et al., 2015) and especially from the central nucleus of the amygdala (CeA) (Tjoumakaris et al., 2003). It is also reported that CRF and glutamate are frequently colocalized in axon terminals arising from CeA to make synaptic contacts onto dendrites of LC neurons (Valentino et al., 2001).

**Figure 3:**
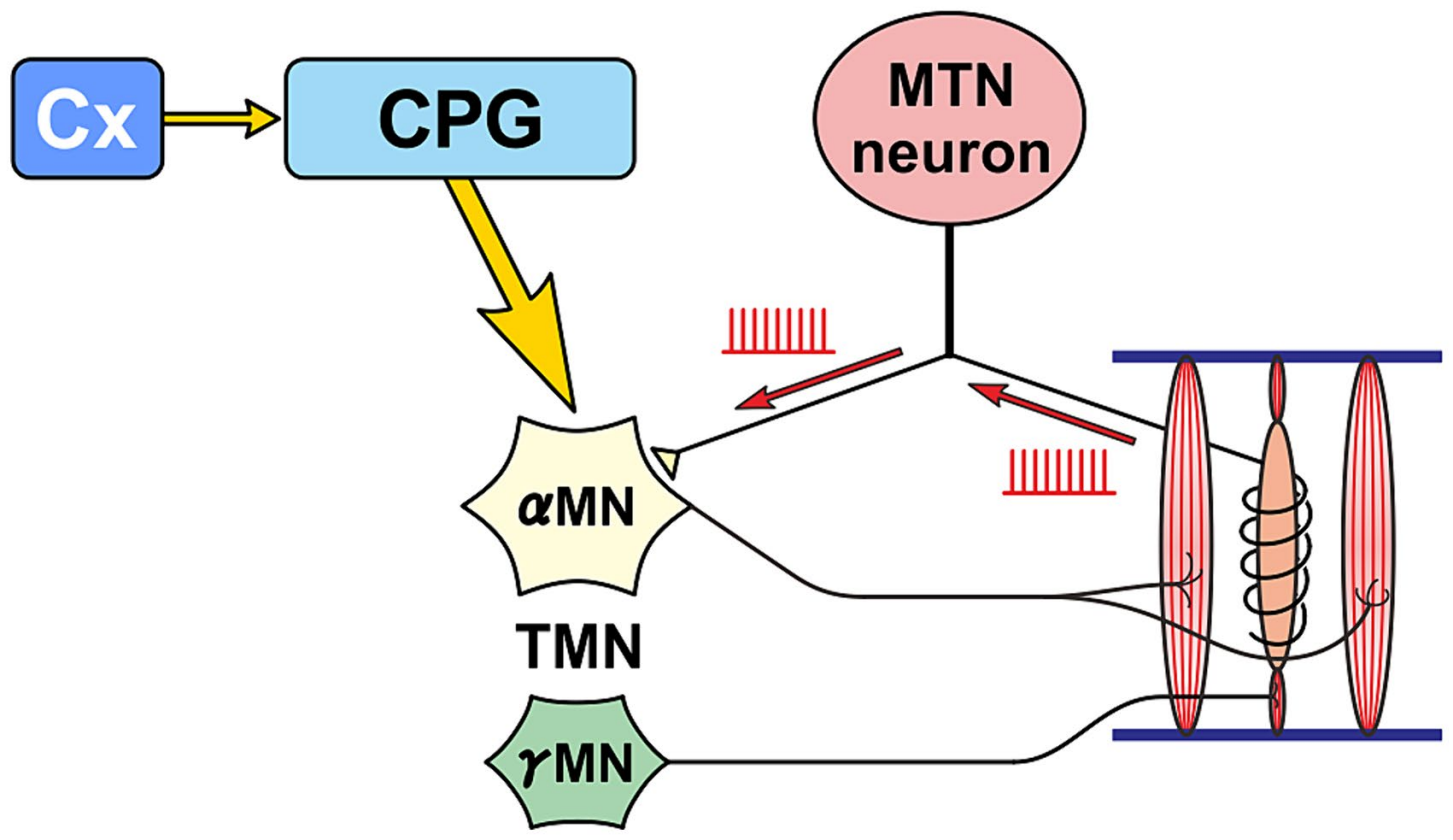
Primary sensory neuron mode. A functional mode that faithfully transmits the impulse activity arising from muscle spindles to α-motoneurons (αMN). Precise masticatory movements are possible.

**Figure 4:**
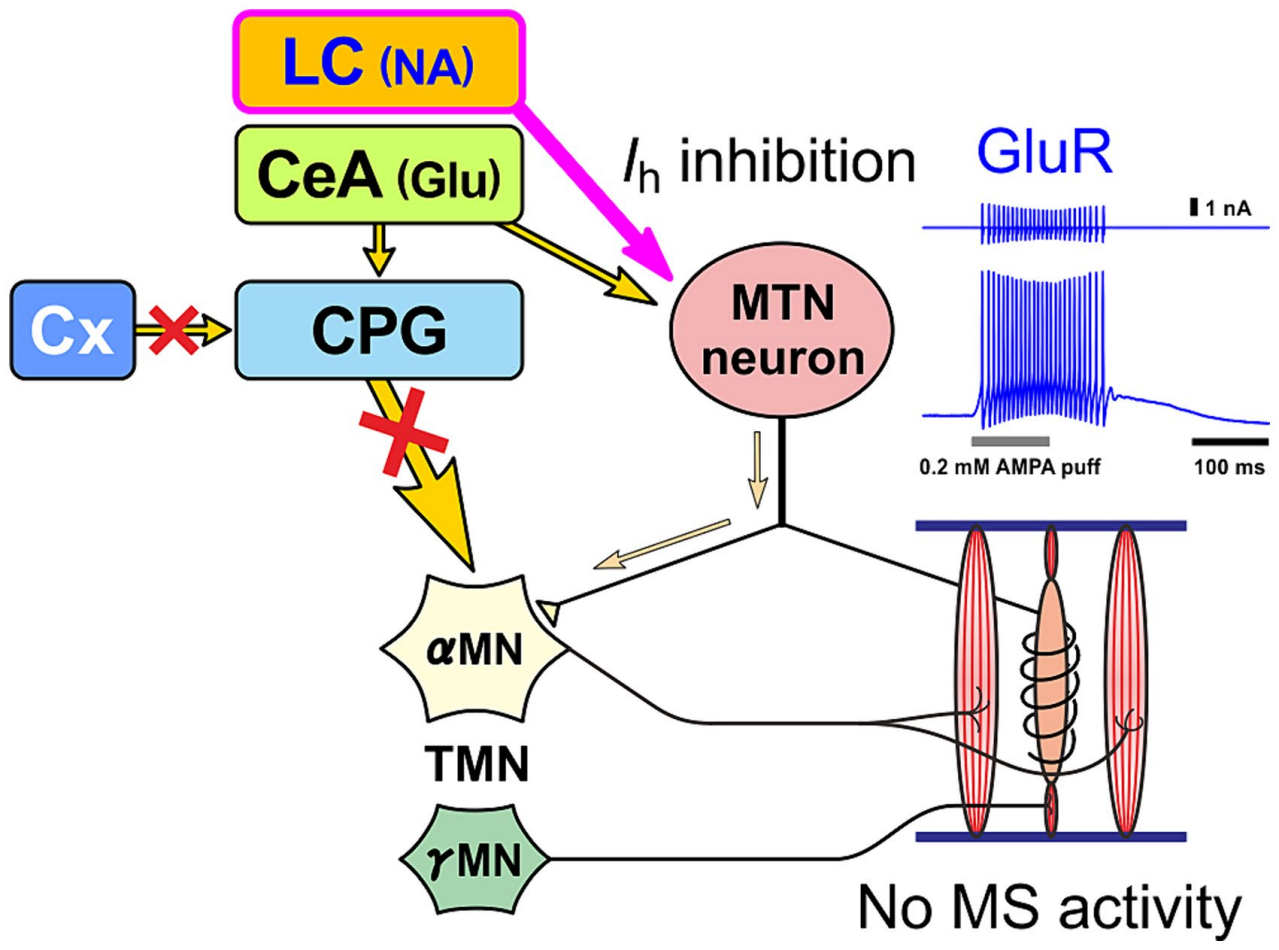
Premotor neuron mode. When MTN neurons receive NA input from LC and glutamatergic input from central nucleus of the amygdala (CeA) at the same time, MTN neurons act as premotor neurons that fire in bursts and thereby powerfully drive αMN, without impulses from muscle spindles (MS), to perform biting attacks and predatory activities. Glutamate receptor (GluR) current is enhanced by inhibition of h-current (*I*_h_) as a result of activation of α2A AR by LC inputs (Kawasaki et al., 2018; Toyoda et al., 2022).

In the case of normal masticatory movement commanded by the cortical mastication area, MTN neurons act as primary sensory neurons by faithfully transmitting information arising from muscle spindles to motor neurons, and are involved in the masticatory motor control (Figure 3). On the other hand, there is another jaw-closing movement that occurs with the involvement of LC (Figure 4), as the neural projections from LC to MTN have already been reported (Copray et al., 1990; Takahashi et al., 2010). Such neural projection would act to induce an attacking-bite against the enemy when aggression is heightened in response to encountering the enemy. It has been shown that stress and aggressive emotions can activate CeA (Haller, 2018) which sends direct excitatory glutamatergic inputs to MTN (Shirasu et al., 2011; Zhao et al., 2022). Then, during an attack-biting, both LC and CeA would be simultaneously activated. Simultaneous activation of these two inputs results in an amplification of glutamatergic currents in MTN neurons by the action of NA-ergic inputs, causing MTN neurons to fire in bursts (Kawasaki et al., 2018; Figure 4). Such burst firings can rapidly trigger and recruit the jaw-closing motoneurons, causing rapid and powerful, i.e., ballistic, jaw-closing movements. The involvement of the CeA in attacking-bite has been reported previously, but a pathway through the parvocellular reticular nucleus to the trigeminal motor nucleus has been proposed (Han et al., 2017). However, the central pattern generator (CPG), which receives input from the masticatory cortex for normal masticatory movements, is also believed to include the parvocellular reticular nucleus (Nozaki et al., 1993). Therefore, further verification is needed to determine whether such a multisynaptic circuit can function as a neural circuit to trigger ballistic biting attacks, which are rapid and powerful non-rhythmic jaw-closing movements, in contrast to normal slow rhythmic masticatory movement.

The biting attack is common to all animal species. For all carnivores, the two most important behaviors are the capture and eating of prey with the mouth and the attacking bite when attacking an enemy. The jaw-closing movements during biting attacks are ballistic movements, and the firing patterns and functions of MTN neurons are different between ballistic and isometric jaw-closing (Tsukiboshi et al., 2012) observed during attacking bite and mastication, respectively (Figures 3, 4). Therefore, vertebrates, including humans, are thought to have developed specialized brain circuits to attain such movements. These may be one of the reasons why the MTN is located in the brain as the only exception in spite of being primary sensory neurons, and why it came to be located lateral to the LC.

## 7 Discussion

The occlusal and masticatory dysfunctions induce stress while stress induces bruxism and clenching, which leads to the temporo-mandibular joint (TMJ) disorder and other occlusal and masticatory dysfunctions, thus causing a negative or vicious circle. When this vicious cycle progresses, TMJ disorder or occlusal and masticatory dysfunctions are believed to result in brain dysfunctions such as depression or learning/memory impairment. Thus, it has been suggested that occlusal and masticatory dysfunctions may be involved in the pathogenesis of dementia. Nevertheless, the neural mechanism linking masticatory dysfunction and such dementia is not known yet.

### 7.1 Modulation of neurotrophic relationship between LC and MTN by functional modes of MTN neurons

**Figure 5:**
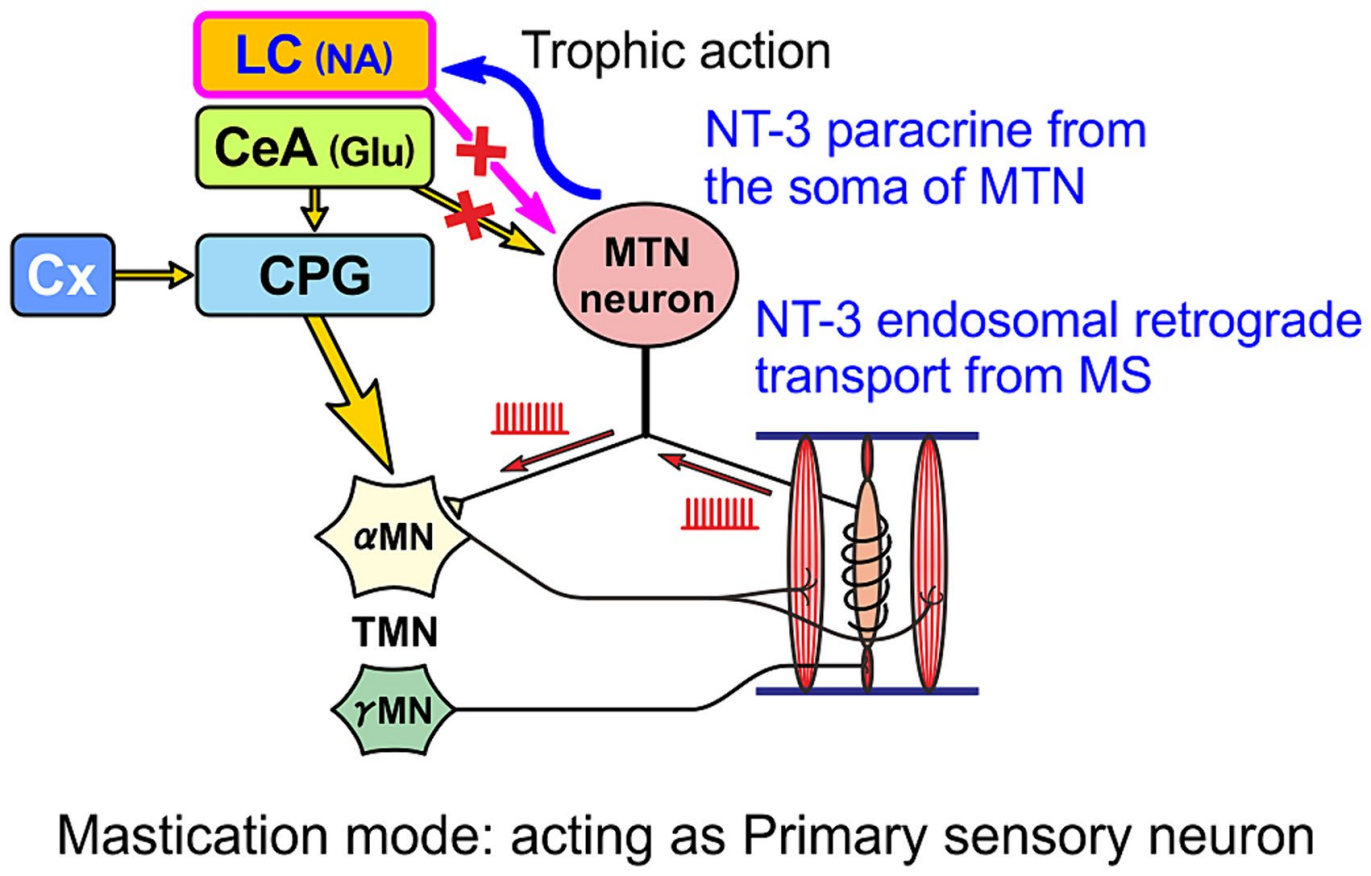
When MTN neurons act as primary sensory neurons, secretory NT-3 produced by muscle spindles (MS) is taken up by endocytosis after binding to TrkC receptors expressed in the axon terminal and transported retrogradely as endosomes through the axon to the cell body of the MTN neuron, from which NT-3 can be further paracrine released to the LC.

As mentioned above, MTN neurons have two functional modes: one mode is to faithfully transmit information from muscle spindles to motor neurons as primary sensory neurons (Figure 3), and the other mode is to act as premotor neurons (Figure 4), which are switched mainly by the action of six different ion channels (Saito et al., 2006; Kang et al., 2007; Chung et al., 2015; Kawasaki et al., 2018). When muscle spindle is most activated by isometric contraction during mastication (as in the primary sensory neuron mode), NT-3 produced by muscle spindle activity is released from it and binds to TrkC receptors expressed on the axon terminal of MTN neurons, and is subsequently taken-up into the axon of MTN neurons as endosomes. Axonal retrograde transport of the endosome of NT-3/TrkC complex occurs to accumulate NT-3 in the cell body of MTN neurons (Figure 5). NT-3 is then further secreted as paracrine molecule from the cell body in response to the increase in [Ca^2+^]_i_ in the cell body caused by firing activity. Although it has been demonstrated electrophysiologically that paracrine secretion occurs in MTN neurons (Zhang et al., 2012), the replenishment of NT-3 may be delayed because the retrograde axonal transport of NT-3 all the way up to the cell body takes time (Chowdary et al., 2012). However, whenever MTN neurons act as a primary sensory neuron mode, NT-3 should be certainly replenished and accumulated in the cell body of MTN neurons (Chowdary et al., 2012).

**Figure 6:**
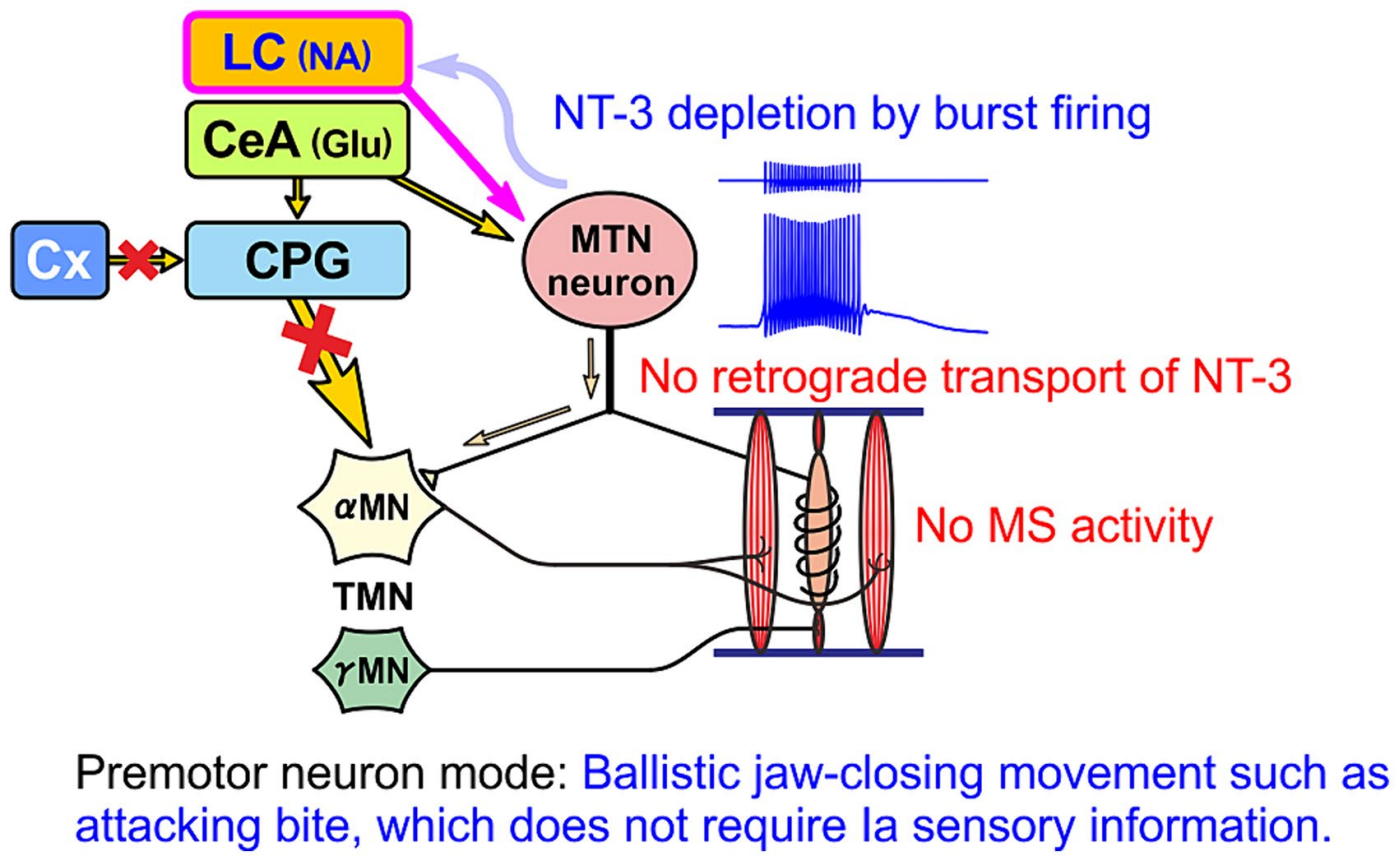
When MTN neurons function as premotor neurons, there is no replenishment of NT-3 from muscle spindles (MS). Then, NT-3 would be finally depleted after secretion of NT-3, without replenishment, by the bursting activity of MTN neurons evoked by coactivation of LC and CeA.

Humans usually do not perform the aggressive attacking-bite. However, there would be a conserved pathway of CeA to MTN that is thought to be involved in causing bruxism (clenching/grinding) under stressful condition (Mascaro et al., 2009). Therefore, in contrast to this primary sensory neuron mode, when stress-induced clenching persists, LC and CeA may be activated and subsequently MTN neurons may continue to function in a premotor neuron mode, firing in bursts. In this case, since there is no activity of muscle spindles, there is no supplementation of NT-3 from muscle spindles and only unilateral paracrine secretion of NT-3, and consequently NT-3 is likely to be depleted in MTN neurons (Figure 6). These notions suggest that the amount of NT-3 accumulated in the cell body and paracrine secretion of NT-3 may differ depending on the functional mode of MTN neurons.

Although various ion channels are involved in switching the activity mode of MTN neurons (Saito et al., 2006; Kang et al., 2007; Chung et al., 2015; Kawasaki et al., 2018), the switching is crucially dependent on the activation of α2A ARs by NA secreted as “volume transmission” from the axon terminal of LC neurons (Toyoda et al., 2022). Since LC neurons can induce burst firing in MTN neurons independently of the activity of the muscle spindles of the jaw-closing muscles required for the precise control of masticatory movements (Kawasaki et al., 2018), it is considered that the activity of LC neurons may interfere with the precise control of masticatory movements by the activity of muscle spindles and may further cause stress. Therefore, when masticatory dysfunction due to such as malocclusion is accompanied by mental stress, the functional relationship including the neurotrophic one between the LC and MTN may be affected, leading to the AD pathogenesis.

We have already mentioned that the paracrine secretion of NT-3 from MTN may affect the maintenance of the function or the survival of LC neurons because MTN medially adjoins LC and receives TrkC-expressing fiber projections from LC neurons (Merlio et al., 1992; Sandell et al., 1994, 1998). Despite the fact that LC and BF are neuronal populations that play a central role in the pathogenesis and progression of AD, there have been very few studies on their maintenance and survival. One study clearly demonstrated that NT-3 suppressed 6-OH-dopamine-induced cell death in LC (Arenas and Persson, 1994) while there have been numerous studies showing that AD pathogenesis is facilitated when BF and LC are lesioned (Laursen et al., 2013; Coradazzi et al., 2016).

### 7.2 Chronic stress can increase NT-3 in LC neurons in a manner independent of muscle spindle activity

Astrocytes or interneurons surrounding the MTN would be activated by glutamatergic inputs from CeA, which primarily activates MTN neurons under stressful conditions. NT-3 release from astrocytes or interneurons in response to glutamatergic inputs (Lessmann et al., 2003; Bronzuoli et al., 2017) may be taken up by TrkC receptors expressed in adrenergic terminals of LC neurons under stress condition. Furthermore, *m*RNA levels of NT-3 in LC neurons are increased under repeated restraint stress (Smith et al., 1995) whereas endogenous expression of NT-3 is absent in normal condition (Akbarian et al., 2001). Thus, only under stress conditions, NT-3 emerges in LC neurons endogenously and exogenously through activation of TrkC receptors. This may be necessary to protect LC neurons from excitotoxicity induced by activation of glutamate and CRF receptors under stress condition, as NT-3 can protect neurons from excitotoxicity presumably by stabilizing [Ca^2+^]_i_ (Cheng and Mattson, 1994; Safina et al., 2015). In spite of such neuroprotective mechanisms, LC neurons are very vulnerable to chronic stress (Sanchez-Padilla et al., 2014; Wang et al., 2020; Evans et al., 2022), suggesting that NT-3 brought about by such mechanisms may not be able to cope with chronic or severe stress.

On the other hand, BDNF in hippocampus is decreased by reduced mastication (Fukushima-Nakayama et al., 2017) or by restraint stress but is restored by active mastication (Lee et al., 2008). Considering the role of NA inputs to hippocampus/cerebral cortex in producing BDNF in those cortices (Counts and Mufson, 2010), those findings suggest that LC neurons under stress would not contribute to the production of BDNF in those cortices whereas active mastication plays a crucial role in maintaining the functional role of LC neurons to produce BDNF in those cortices. Thus, in terms of neuroprotective action through activation of β AR in those cortices (Counts and Mufson, 2010), higher frequency phasic firing in LC neurons under stress condition may not be so effective than slow tonic firing under normal condition, as reported in the signal transduction in α2A ARs previously (Toyoda et al., 2022). NT-3 produced by muscle spindle activity may be useful to maintain normal neuronal activity of LC neurons while under severe stress condition NT-3 would be necessary to just protect LC neurons from excitotoxicity. Nevertheless, persistent overexcitation of LC neurons under chronic stress would lead to their degeneration due to the endogenous production of 3,4-dihydroxyphenylglycolaldehyde (DOPEGAL) and tau protein.

### 7.3 Endogenous mechanism of cell death of LC neuron in AD and its modification by NT-3

In LC neurons, NA is metabolized by mitochondrial monoamine oxidase A (MAO-A) to produce DOPEGAL. Since DOPEGAL produces free radicals, its cytotoxicity has been studied extensively (Burke et al., 1999; Kang et al., 2020). Recently, it was shown that DOPEGAL activates asparagine endopeptidase, resulting in the production of hyperphosphorylated tau protein and Aβ (Kang et al., 2020) and leading to AD (Kang et al., 2020, 2022). However, why and how free NA accumulates in the cell bodies of LC neurons has not been questioned. Until this is clarified, it remains unclear why DOPEGAL is overproduced in the cell body (Burke et al., 1999) and whether it can be the earliest pathogenesis of AD. Normally, free NA is internalized into vesicles by vesicular monoamine transporter 2 (vMAT2) rather than metabolized by MAO-A into DOPEGAL, and vesicles containing NA are paracrine secreted from the cell body upon neuronal activity. This is because vMAT2 has a higher affinity for NA than MAO-A has (Chaudhry et al., 2008; Meiser et al., 2013). Thus, the mechanism by which NA accumulates in the cell body is still unknown and has not ever been focused on.

It is also not known yet how NT-3 modulates these endogenous mechanisms of LC degeneration. Stress and/or glucocorticoid exposures lasting 24 h or longer have been associated with greater MAO-A levels/activity (Filipenko et al., 2002; Ou et al., 2006). NT-3 can protect neurons from excitotoxicity presumably by stabilizing [Ca^2+^]_i_ (Cheng and Mattson, 1994; Safina et al., 2015). Because MAO-A is known to be activated by [Ca^2+^]_i_ increase (Cao et al., 2007), it would be interesting to examine whether activity of MAO-A is suppressed by NT-3 or not.

## Author contributions

YK: Conceptualization, Funding acquisition, Writing – original draft, Writing – review & editing. HT: Writing – review & editing. MS: Conceptualization, Writing – original draft, Writing – review & editing.
